# Wear performance of metal and ceramic long-neck femoral head taper connections: an experimental ex vivo study

**DOI:** 10.2340/17453674.2026.46301

**Published:** 2026-08-13

**Authors:** Margarida RIBEIRO de SOUSA, Perttu NEUVONEN, Vesa SAIKKO

**Affiliations:** 1 Aalto University, Espoo; 2 Coxa Hospital for Joint Replacement, Tampere, Finland

## Abstract

**Background and purpose:**

The wear performance of femoral head taper connections is important for the longevity of total hip replacement. Metal release may cause adverse tissue reactions. There are some clinical indications that long neck heads may show increased wear, but no laboratory tests have been published. We aimed to examine the wear performance of femoral head taper connections in long and extra-long neck heads.

**Methods:**

Load frame and hip joint simulator wear tests were performed with the same stem designs as in the reference study. The CoCr and zirconia toughened alumina (ZTA) heads of 36 mm diameter were of the long or extra-long neck type. Optical and scanning electron microscopy were utilized to study wear marks, and mass spectrometry to estimate Ti and Co release. Head disassembly forces were measured.

**Results:**

Our preclinical, design-specific results showed that, compared with medium necks, long and especially extra-long necks led to increased wear while maintaining an asymmetric wear pattern more widely distributed throughout the contact area. In the hip joint simulator tests, 1 neck fracture occurred.

**Conclusion:**

The long and extra-long head taper connections showed increased wear compared with that of medium heads. Although no serious damage occurred, CoCr head tapers were the most affected compared with ZTA.

Modularity of femoral heads allows the use of different materials, diameters, and neck lengths for specific needs in total hip arthroplasty. The taper connection of the femoral head is an important interface because its wear performance may affect the longevity of the implant [1]. The interface is responsible for the load transition between the femoral head and femoral stem. Metal release from the interface may cause adverse tissue reactions [2]. In an earlier study, the wear performance of femoral head taper connections with medium neck lengths of 2 widely used designs was evaluated experimentally [3]. The wear marks were relatively mild. There are some clinical indications that wear increases with increasing neck length [4,5]. It has been suggested that the increased bending moment at the connection, referred to as the toggling effect, can be harmful for the taper wear performance [6,7]. Therefore, the wear behavior of long neck heads was considered a relevant topic, even though long neck heads are used less frequently than medium heads. No laboratory studies on the wear performance of long neck heads have been published.

We aimed to examine the possible differences in wear produced with different neck length and material. The principal research question was: is the wear of long neck head taper connections, compared with that of medium necks, a cause for concern?

## Methods

### Study design

This was an experimental study using a load frame test based on ISO 7206-6 and an ISO 14242-1 hip joint simulator to assess the femoral head taper connection wear of 36 mm CoCr and ZTA long/extralong heads paired with Ti-6Al-4V femoral stems from 2 contemporary designs. The study included microscopy, ICP-MS measurements, and the measurement of disassembly forces. No human subjects were involved (ethical approval not required).

The study is reported according to the CRIS guideline.

### Materials

**Figure 1:**
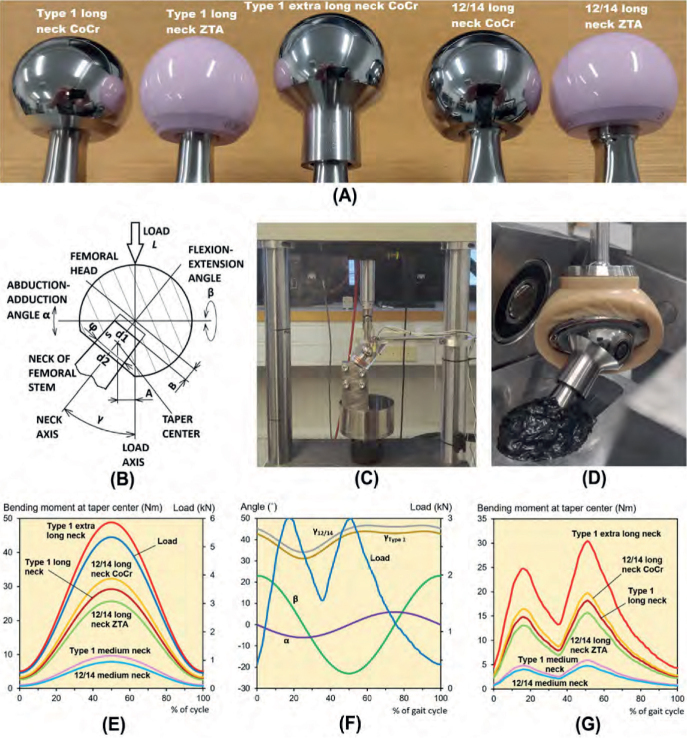
(A) Femoral heads on stems. (B) Taper connection with long neck length femoral head (see Table 2). (C) Load frame (LF) test ongoing with Type 1 extra-long neck CoCr head. (D) Hip joint simulator (HJS) test setup with Type 1 extra-long neck CoCr head shown without lubricant chamber. (E) Variation with time of LF test load *L* and bending moment M at taper center of 6 forms of taper connections (see Table 2), cycle time = 50 ms. (F) Variation with time of HJS test load *L*, abduction–adduction α, flexion–extension β, neck axis to load axis angles γ_Type 1_ = arccos(cos(37˚+α)cosβ) and γ_12/14_ = arccos(cos(40˚+α)cosβ), gait cycle time = 0.94 s. (G) Variation with time of HJS test bending moment at taper center, M = *L*×A, where A = (s/2+B)sinγ.

Ti alloy femoral stems and long neck CoCr and zirconia toughened alumina (ZTA) femoral heads of 36 mm diameter were purchased from the Coxa hospital stock (Figure 1A, Table 1). The stems were Taperloc Complete with Type 1 Taper (20 × 160 mm Standard Offset, Zimmer Biomet, Warsaw, IN, USA) and Summit with 12/14 Taper (Size 10 STD 170 mm, DePuy Synthes, Warsaw, IN, USA). With all heads, the entire taper contact was distal to the head center (Figure 1B, Table 2).

**Table 1:** Specimens

Design	Component	Manufacturer’s taper designation	Catalog #	n
Taperloc Complete	Ti alloy stem	Type 1 Taper	51-103200	18
	CoCr head	Type 1 Taper, +6 mm neck	11-363664	6
	ZTA head	Type 1 Taper, +6 mm neck	650-0663	6
	CoCr head	Type 1 Taper, +12 mm neck	11-363666	6
Summit	Ti alloy stem	12/14 Taper	1570-01-180	12
	CoCr head	12/14 Taper, +12	1365-54-000	6
	ZTA head	12/14 Taper, +12	1365-36-340	6

**Table 2:** Measurements and calculations for 4 forms of taper connections tested with LF and HJS (see Figure 1B)

Taper connection	d1 ^a^ (mm)	d2 ^b^ (mm)	s ^c^ (mm)	ȹ ^d^ (˚)	B ^e^ (mm)	γ_LF_^f^ (°)	A_LF_^g^ (mm)	M_LFmax_^h^ (Nm)	γ_HJS50%_^i^ (°)	A_HJS50%_^j^ (mm)	M_HJSmax_^k^ (Nm)
Type 1 long neck CoCr, ZTA	11.50	12.06	8.4	3.8	4.7	37.9	5.5	29.2	42.7	6.0	18.1
Type 1 extra-long neck CoCr	11.50	12.06	8.4	3.8	10.7	37.9	9.2	48.9	42.7	10.1	30.3
12/14 long neck CoCr	12.61	13.61	10.5	5.5	4.0	40.8	6.0	32.3	45.2	6.6	19.7
12/14 long neck ZTA	12.61	13.25	6.7	5.5	4.0	40.8	4.8	25.7	45.2	5.2	15.6

a Minimum diameter of contact, measured with digital caliper from trunnions. Range of variation ±0.01 mm.

b Maximum diameter of contact. measured with digital caliper from trunnions. Range of variation ±0.01 mm.

c Contact length, measured with digital caliper from trunnions. Range of variation ±0.1 mm. With 12/14 ZTA head, contact was shorter than with 12/14 CoCr head (see Figure 1A).

d Taper angle = 2arcsin((d2-d1)/2s). Range of variation ±0.1º.

e Distance from head center to proximal end of taper contact that was distal to head center, whereas with medium heads, it was proximal to head center [3]. Evaluated using digital height gauge. Range of variation ±0.1 mm.

f Neck axis to load axis angle in LF tests = arccos(cosγ_β=0˚_cos9˚). γ_β=0˚_ was 37˚ with Taperloc and 40˚ with Summit, as their neck axis to distal stem axis angles were 133˚ and 130˚, respectively, and ISO 7206-6 specifies that adduction of distal stem axis = 10˚, and that β = 9˚. Estimated range of variation ±0.5º.

g Moment arm of load relative to taper center in LF tests = ((√(s^2^–((d2–d1)/2)^2^)/2)+B)sinγ_LF_ ≈ (s/2+B)sinγ_LF_. Estimated range of variation ±0.2 mm.

h Bending moment maximum at taper center in LF tests = 5.34 kN × A_LF_. Estimated range of variation ±1 Nm.

i Neck axis to load axis angle in HJS tests at 50% of gait cycle = arccos(cos(γ_β=0˚_+0˚)cos(–23˚)) (see Figure 1F). Estimated range of variation ±0.5º.

j Moment arm of load relative to taper center in HJS tests at 50% of gait cycle ≈ (s/2+B)sinγ_HJS50%_. Estimated range of variation ±0.2 mm.

k Bending moment maximum at taper center in HJS tests that occurred at 50% of gait cycle = 3.0 kN × A_HJS50%_. Estimated range of variation ±1 Nm.

### Experimental setup

The moment arms A of the load L relative to the taper center in Taperloc’s “Type 1 Taper, +6 mm neck” and Summit’s “12/14 Taper, +12” heads were close to each other despite the different neck length designations by the manufacturers. Hence, both of them are hereafter systematically called, for simplicity, long neck heads. An extra-long neck head was available only for Type 1 CoCr. This was added to the study as a “worst case.” The heads were assembled by an impact force of 2.3 kN [8].

### Tests

The 2 test methods were similar to those of an earlier study with medium neck length heads [3]. The present study did not include a medium neck control group. All specimens belonged to the long neck configurations already described and any comparisons with the earlier medium neck results are external and observational. In all tests, the sample size chosen was 3, which is generally considered to be the minimum statistically, and here limited mainly by the available resources. Briefly, the 2 test methods were as follows.

Load frame (LF) tests based on ISO 7206-6 [9] applied a sinusoidal load cycle, 0.55 to 5.34 kN, vertically to the femoral head 20 times per second for 10 million cycles (Figures 1C and 1E, Table 2). The direction of load relative to the taper connection was stationary. The test environment was 0.9% NaCl in deionized water at 37±2˚C. ISO 7206-6 parameters were used because there is no ISO standard specifically for the wear testing of the taper connection. ISO 7206-6 is primarily an endurance test for the neck regarding fatigue, and therefore it was considered sufficiently demanding also for the present purpose. The high frequency that was readily attained was a substantial advantage for the execution of the test program.

Hip joint simulator (HJS) tests, based on ISO 14242-1 [10,11] simulated level walking for 5 million cycles at 1 cycle/s with a double-peak (3.0 kN) load (Figures 1D, 1F, and 1G, Table 2). The articulation, against a Vivacit-E liner, was multidirectional. The direction of load relative to the taper connection varied continuously. In addition, the articulation friction affected taper connection stresses. The test environment was diluted alpha calf serum (protein concentration 20 mg/mL) at 37±2°C, replaced every 0.625 million cycles. HJS wear tests for the taper connection using actual femoral stems represented a scientific novelty. Hence, LF tests served also as a reference for the HJS tests in the sense that in biomechanical research load frames are much more common than hip joint simulators.

### Measures

**Figure 2:**
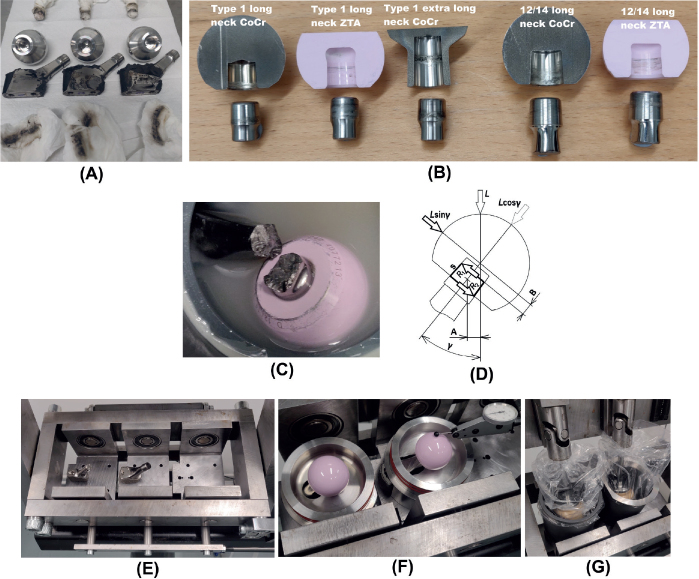
(A) Type 1 extra-long neck specimens after HJS test, note sludge from taper connections on tissue papers. (B) Specimens from HJS test cut for microscopy, superior surfaces. (C) Fracture of Summit stem with long neck ZTA head in HJS test at 2.8 million cycles. (D) Schematic of “toggling.” Simplified, R_1_ and R_2_ were resultant forces of compressive stress concentrations due to bending moment at taper center M = L×A = (s/2+B)*L*sinγ, where *L*sinγ was radial component of *L* (see Figure 1B and Table 2). Head equilibrium resulted in R_1_ = ((s+B)/s)*L*sinγ and R_2_ = (B/s)*L*sinγ. In HJS tests, M_max_ = *L*_max_×(s/2+B)sinγ_HJS50%_. With 12/14 long neck ZTA head, M_max_ = 15.6 Nm, R_1_ = 3.40 kN and R_2_ = 1.27 kN. With 12/14 long neck CoCr head, corresponding values were 19.7 Nm, 2.94 kN, and 0.81 kN. The ZTA head had 36% shorter contact and 20% lower bending moment but 16% larger R_1_ and 56% larger R_2_, which could influence wear. Assembly load and axial component of load, *L*cosγ, caused axisymmetric stresses and were omitted here. (E) New specimen holders enable use of all 12 test stations of HUT-4 HJS concurrently in taper connection wear tests with proximal stems first fixed to new base plates of abduction-adduction cradles. (F) stainless steel bases fixed and heads aligned, and (G) stems sealed with polyurethane sealant, o-rings, lubricant chambers, lubricant, acetabular components, loading bars, and plastic covers added.

After the tests, the heads were disassembled so that the force required was measured, the taper surfaces were cleaned, and the specimens were cut for optical and scanning electron microscopy (SEM) (Figures 2A and 2B). For wear regime identification and general visual assessment, the specimens were analyzed using a Zeiss Stemi 508 Stereo Microscope and a Zeiss Merlin Field Emission Scanning Electron Microscope (SEM) equipped with a Bruker Quantax Energy Dispersive Spectrometer (EDS), which was used for the Energy Dispersive X-ray Spectroscopy (EDX) analysis (Carl Zeiss Microscopy GmbH, Jena, Germany). For SEM analysis, the ZTA femoral heads were coated using a Leica EM ACE600 carbon-thread evaporator and sputtering system with an Au/Pd layer approximately 8 nm thick. Corrosion and fretting damage at the taper connections were assessed based on the morphology and extension of the damage following the Goldberg scoring system [12].

Vickers macrohardness (HV10) of femoral stems was measured using a Brickers 220 hardness tester (Gnehm, Switzerland). For gravimetric evaluations of femoral heads, a Mettler AT261 DeltaRange and a Mettler Toledo XSR105 DualRange balances were used (Mettler-Toledo, Greifensee, Switzerland).

To obtain the concentrations of the most essential elements, Ti and Co, pooled saline and serum samples from each test were analyzed by means of inductively coupled argon plasma mass spectrometry (Thermo iCAP RQ ICP-MS, Thermo Fisher Scientific Inc, Waltham, MA, USA) by the Finnish Institute of Occupational Health. All solutions were prepared using ultra purified water (18.2 MΩ-cm). All single element and multielement standards were ICP-MS grade and supplied by Merck KGaA (Darmstadt, Germany), Romil, Ltd (Cambridge, UK), and VWR (International LLC). In-house laboratory spiked controls, commercially available controls (ClinChek Urine Control, lyophil., for Trace Elements, Level I, II), and external quality controls (German External Quality Assessment Scheme, G-EQUAS, Institute and Out-Patient Clinic for Occupational, Social and Environmental Medicine of the University Erlangen-Nuremberg, Germany) were used as quality controls. For the estimation of the total release, the Ti and Co concentrations were multiplied by the total volume of fluid used in each test.

### Ethics, data sharing, funding, and disclosures

The study did not involve human subjects. Therefore, ethical approval by an ethical review board was not needed.

Data produced in the present study is available on the Zenodo repository, https://doi.org/10.5281/zenodo.18953264. The study was funded by the Research Council of Finland (Scientific Council for Natural Sciences and Engineering, Grant No. 355734) and Aalto University.

PN reports grants from Pirkanmaa hospital district, during the conduct of the study. VS reports grants from Research Council of Finland, during the conduct of the study. The authors declare that they have no competing interests. Complete disclosure of interest forms according to ICMJE are available on the article page, doi: 10.2340/17453674.2026.46301

## Results

**Figure 3:**
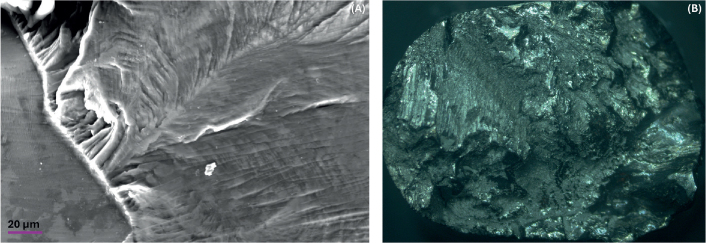
Fracture surface of Ti‑6Al‑4V femoral stem. (A) SEM micrograph showing river marks emanating from origin and increasing surface roughness away from it, (B) stereomicroscopy image of entire fracture topography.

The tests were uneventful with one exception. The neck of a Summit stem fractured with a long neck ZTA head in an HJS test at 2.8 million cycles (see Figure 2C). Fractography revealed a relatively smooth region with distinct river mark, microscopic lines that merge and point to the fracture origin, from which the surface roughness visually increased with increasing distance (Figure 3), while dimples, characteristic of ductile overload, were not observed. Vickers hardness values for fractured and non‑fractured Summit stems were similar, 330 to 340 HV10.

### Wear

**Figure 4:**
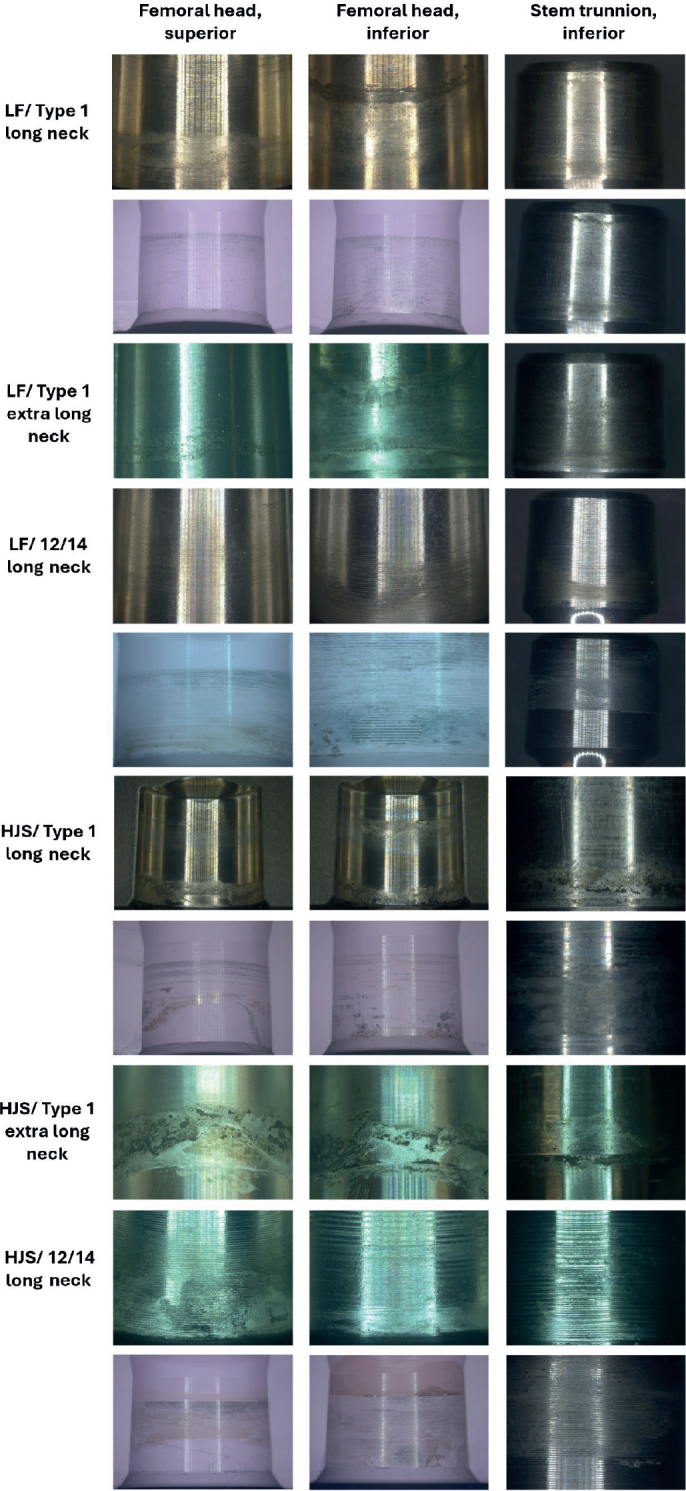
Stereomicroscope images of taper surfaces.

**Figure 5:**
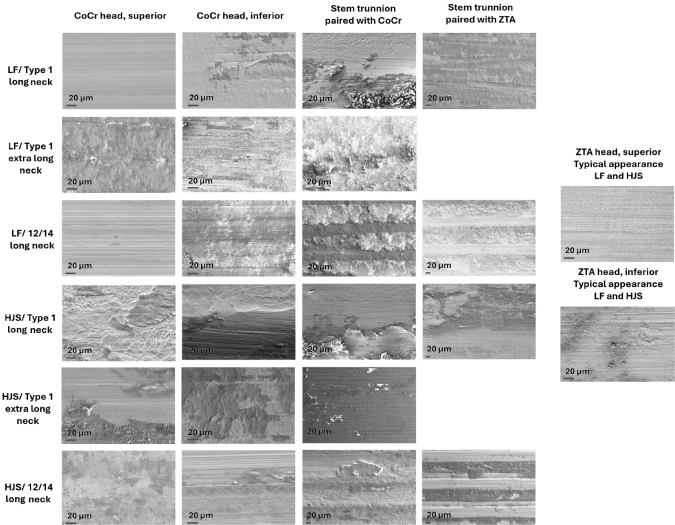
SEM micrographs of taper surfaces.

Regarding the long vs medium neck behavior, Ti and Co releases with long and extra-long neck heads were usually higher than those measured for the medium neck head tests (Table 3). The large difference in Ti vs Co release was conspicuous, as Ti release was in the µg range, 0.03 to 37 µg, and Co release was in the mg range, 0.4 to 2.4 mg. For qualitative analysis and comparison between the medium neck data, stereomicroscope analysis documented the surface features of the long neck heads (Figure 4). Long and especially extra-long neck CoCr heads showed broader wear marks, mainly on the distal and inferior regions, consistent with patterns reported for medium neck lengths, but spreading further circumferentially across the contact area. For a qualitative context, the previous medium neck study [3] showed predominantly inferior–distal wear with limited circumferential spread on CoCr heads, and fewer metal transfer spots on ZTA heads. On the ZTA heads, discrete darker deposits consistent with metal transfer were more numerous and more widely distributed than with medium necks. These results were verified using SEM (Figure 5), which revealed the surface microstructure of the specimens. The transfer was visually striking but the magnitude of transferred Ti alloy was below 0.1 mg, as determined by the weighing of the heads.

**Table 3:** Titanium and cobalt release, mean (SD)

Test	Taper	Femoral head material	Ti (*µ*g)	Co (*µ*g)
LF	Type 1 medium neck**^a^**	CoCr	3.50 (0.11)	1,300 (40.4)
		ZTA	0.08 (0.01)	
	12/14 medium neck	CoCr	0.85 (0.01)	355 (9.59)
		ZTA	0.68 (0.08)	
	Type 1 long neck	CoCr	0.54 (0.03)	440 (17.4)
		ZTA	0.03 (0.00)	
	Type 1 extra-long neck	CoCr	3.30 (0.04)	689 (16.1)
	12/14 long neck	CoCr	4.31 (0.08)	567 (7.13)
		ZTA	6.42 (0.45)	
HJS	Type 1 medium neck	CoCr	14.2 (1.52)	1,760 (161)
		ZTA	8.34 (0.35)	
	12/14 medium neck	CoCr	14.9 (0.85)	639 (32.5)
		ZTA	8.00 (0.72)	
	Type 1 long neck	CoCr	17.7 (1.77)	1,670 (51.5)
		ZTA	36.8 (3.86)	
	Type 1 extra-long neck	CoCr	12.9 (1.49)	2,080 (28.8)
	12/14 long neck	CoCr	15.4 (1.49)	2,380 (126)
		ZTA	18.5 (0.95)	

a Medium necks were tested in [3].

### CoCr vs ZTA

Comparing the CoCr vs ZTA behavior, we found that the mean disassembly force of CoCr heads was always larger than that of the corresponding ZTA heads. The disassembly forces of CoCr heads ranged from 1.6 kN to 6.7 kN, and those of ZTA heads from 1.2 kN to 4.2 kN (Table 4). Weight changes of the femoral heads were not measured systematically, but a typical weight loss of CoCr heads was 5 mg, including wear and corrosion, whereas for ZTA heads, no weight change was detected with ±0.1 mg accuracy. The inferior surface of the CoCr head tapers exhibited visually increased surface roughness and more wear marks compared with the superior surfaces. The ZTA head taper surfaces appeared comparatively smooth. In the SEM imaging of the inferior ZTA head taper (see Figure 5), discrete darker spots that could fit with metal transfer, but no material loss, were seen. On the other hand, CoCr head tapers displayed more extensive wear marks across the contact area.

**Table 4:** Femoral head disassembly force, mean (SD)

Test	Taper	Femoral head material	Force (kN)
LF	Type 1 long neck	CoCr	4.53 (0.23)
		ZTA	1.92 (0.26)
	Type 1 extra-long neck	CoCr	6.13 (0.58)
	12/14 long neck	CoCr	1.73 (0.18)
		ZTA	1.43 (0.28)
HJS	Type 1 long neck	CoCr	5.48 (0.21)
		ZTA	3.42 (0.76)
	Type 1 extra-long neck	CoCr	5.49 (0.28)
	12/14 long neck	CoCr	3.55 (0.25)
		ZTA	2.58 (0.20)

### Taper type

Between Type 1 and 12/14, design-related differences were observed regarding surface features and Goldberg scores [12]. SEM analysis showed that the wear of the 12/14 stem trunnions matched with that of their corresponding CoCr femoral head tapers in both the LF and HJS tests. The trunnion surfaces showed smoothing of the ridges, as well as localized microcracking and darker regions between the ridges. For CoCr long neck femoral heads paired with 12/14 trunnions, imprinting features were visible consistently on the inferior half of the head taper surfaces, independent of the test conditions. These features were absent with the smooth Type 1. Following the Goldberg scoring system [12], including both SEM and stereomicroscopy, LF Type 1 long neck configuration was scored as corrosion 2 (mild) and fretting 2, minor signs of discoloration, mainly on the trunnion, and some localized fretting marks. LF 12/14 long neck was evaluated corrosion 1 (none) and fretting 2, showing no sign of discoloration but visible fretting aligned with the trunnion ridges and their imprint onto the head taper surface. HJS Type 1 long neck was graded corrosion 1 and fretting 2, as this combination showed some wear and roughened topography on the superior and inferior parts of the head taper with scattered fretting on the trunnion as well. HJS 12/14 long neck was assigned corrosion 2 and fretting 2, characterized by surface discoloration mainly on the trunnion and mild fretting on the CoCr head taper. HJS Type 1 extra-long neck was scored corrosion 2 and fretting 2, as there was some localized surface discoloration and visible fretting marks. Finally, the LF Type 1 extra-long configuration also showed corrosion 1 and fretting around 2 or 3 (moderate), attributed due to no visible discoloration but roughened topography on the superior and inferior parts of the head taper with scattered fretting on the trunnion as well, to a slightly greater extent than that of the HJS Type 1 long neck combination.

HJS tests, as compared with LF tests, typically showed higher Ti and Co releases. SEM imaging showed that HJS specimens demonstrated substantially more wear marks than the LF specimens, particularly on the superior halves of the CoCr femoral head tapers that appeared nearly smooth with minimal wear marks. Both trunnion designs paired with the CoCr femoral heads from the HJS test showed wear marks and roughened topography on the superior regions of the tapers.

## Discussion

We aimed to examine wear performance of femoral head taper connections in long and extra-long neck heads. We showed that the wear performance of long and extra-long head taper connections showed increased wear to that of medium heads. Although no serious damage occurred, CoCr head tapers were the most affected compared with ZTA. The increasing neck length led to a broader circumferential distribution of fretting at the taper junction, converting the predominantly inferior–distal wear pattern seen with medium neck lengths into a slightly more distributed one while preserving the same asymmetry.

Our findings on the broadened circumferential fretting distribution with preserved inferior–distal asymmetry agreed with earlier computational studies [13]. The increased wear was especially noticeable on the CoCr femoral heads and their corresponding trunnions. The 12/14 tapers showed ridge imprinting and more corrosion than Type 1. Extra-long neck CoCr heads visually showed the most wear, with HJS overall producing more wear than LF. CoCr heads showed a typical weight loss of 5 mg, while ZTA heads had no detectable weight change. This was in agreement with clinical observations [14].

Clinically, the taper connection performance of DePuy’s ASR XL large-diameter metal-on-metal design was exceptionally poor. The mean wear rate of the CoCr head taper was found to be 3.7 mg/year (range 0.2 to 70 mg/year; volumetric values converted using CoCr density 8.4 mg/mm^3^) [7]. The present CoCr heads showed a typical taper wear of 5 mg in 5 million cycles in the HJS tests. 2 million cycles is usually taken to correspond to 1 year in vivo [15]. Hence, the present wear rate could be taken to correspond to 2 mg/year. This is of the same order of magnitude as the mean reported by Langton et al. [7] and is therefore likely to have clinical significance. In the same study, Langton et al. identified a large value of the so-called horizontal lever arm (HLA), which was considered to be an important factor behind the taper failure. Although the definition of the HLA is not the same as that of the present moment arm A, the detrimental effect is likely to be similar.

In another hip joint simulator study, the mean taper wear of 32 mm diameter CoCr heads against custom-made Ti alloy trunnions, which mimicked DePuy’s 12/14 Corail taper, was 1.8 mg after 5 million cycles [16]. This is substantially lower than the corresponding value in the present study, 5 mg, possibly for the following 2 reasons. First, the peak load in [16] was 2 kN, whereas 3 kN was used in the present study. Second, an assembly load of 4 to 5 kN was used in [16], but only 2.3 kN in the present study, based on cadaver studies [8]. The “optimal” value of 4 to 5 kN, recommended by manufacturers, which appears to be difficult to attain in the surgery [8], may effectively reduce micromotion and consequent wear. The effect of assembly load on wear would be a most interesting topic in future studies.

The observed distribution of wear marks indicated asymmetric contact conditions within the taper junction with more wear on the inferior regions of the CoCr tapers and the trunnions. This asymmetry, which outweighed the possible angular mismatch effect [17], was consistent with the larger bending moment due to the increased neck length, which may promote localized contact stresses and increased micromotion. In comparison, the smooth surface morphology of the ZTA femoral head tapers indicated superior wear resistance in comparison with the CoCr components. The minor darker regions observed on the ZTA head tapers were consistent with metal transfer from the trunnions, in agreement with [6]. Because ZTA heads exhibited no detectable mass change (< 0.1 mg), the total mass of transferred material associated with the dark deposits was below 0.1 mg. In addition, the minor roughness observed on ZTA tapers may be a product of the conductive coating applied for SEM imaging. Both the stereomicroscope and SEM images (see Figures 4 and 5) of the CoCr head taper (HJS, 12/14) confirmed the presence of localized mechanical wear, including fretting and adhesive wear. The images also revealed plastic deformation and smoothing of the indentation grooves. The dull regions in the stereomicroscope image suggested surface roughening. The imprinting on the CoCr heads paired with the 12/14 trunnions and localized polishing on the surface of these trunnions and smoothing of the trunnion ridges was in agreement with the previous work [3], as well as with other published studies [18], and suggested that the taper junction was more prone to gradual surface modification and plastic deformation over time than to severe material loss. The wear marks on the superior halves of the CoCr tapers from the HJS test suggested local contact stresses and micromotion higher than those in the LF tests due to multidirectional loading conditions. In contrast, the near smooth counterparts from the LF tests were consistent with mainly plastic deformation. Both stereomicroscope and SEM imaging confirmed localized fretting, adhesive wear, and plastic deformation for the CoCr heads from the HJS tests regardless of the stem design. This was an indication that the HJS test was more severe than the LF test. The phenomenon of increased micromotion and wear due to asymmetric contact stresses caused by increased bending moments has been called toggling [6] (Figure 2D). Overall, there was a larger micromotion area, consistent with higher bending moments, which confirmed that long necks were more susceptible to taper damage and metal transfer. Our findings were in agreement with the few available clinical observations [4,5].

The largest disassembly forces, 4.3 to 6.7 kN, were measured with the Type 1 smooth tapers with the lower taper angle of 3.8º, and CoCr heads. These taper connections tightened considerably in the tests, as the assembly force was 2.3 kN. The disassembly forces with ZTA heads were always lower than those with the corresponding CoCr heads, as in [3].

The fracture of a Summit stem with a long neck ZTA head in an HJS test (see Figure 2C) was quite unexpected because it occurred (i) with the thicker neck of the 2 types of stems studied, (ii) in the HJS test with a lower peak load of 3.0 kN, and (iii) with a taper connection that had the shortest neck (s/2+B) of the 4 different types studied. A thorough fractography using SEM did not provide an explanation. Although we have high confidence in our testing methods, it cannot be stated with absolute certainty whether the cause of the fracture was a fault in the testing or in the prosthesis. Clinically, the fracture risk of contemporary stems is estimated to be < 0.1% to 3.4% [19].

According to the ICP-MS analyses, an increased neck length usually led to increased metal release, although the difference was not quite as obvious as we had expected. This was probably affected by the low number of specimens available. Compared with the LF tests, HJS tests led to noticeably higher metal release. The fact that in CoCr/Ti alloy interfaces it is CoCr that wears more is explained by the formation of the hard titanium oxide layer that protects the Ti trunnion and is abrasive towards the CoCr counterface [20]. In the HJS test, the direction of loading relative to the taper connection changed continuously, and this possibly led to a less stable, continuously reforming titanium oxide layer and higher Ti release compared with the fixed-direction LF test.

### Strengths

We investigated 2 widely used designs with the most used 2 head materials and the most used head diameter utilizing 2 rigorous test methods based on ISO standards [9,10]. In addition, we used a realistic value for the assembly force of the head and a common liner material and a common acetabular abduction angle in the HJS tests. The head disassembly force was also measured according to ASTM F2009. Moreover, our microscopy and ICP-MS methods represented state of the art. As very little has been published on the performance of the taper connections of long neck heads, our study helped to remedy this shortcoming. These facts increased the clinical relevance of our study.

### Limitations

The HJS test length corresponded to 2.5 years in vivo only [15]. Nevertheless, considerable wear was produced as the tests resulted in measurable damage. Co release was in the mg range whereas Ti release was in the µg range (see Table 3). Goldberg scoring indicated corrosion in the 1 to 2 range and fretting from 2 to 3. Moreover, we had to settle for the minimum sample size of 3 and so the power of the tests was admittedly low. Microscopically, differences in appearance between different taper connections could be detected even with n = 3, the variation within a sample taken into account. The mentioned limitations were due to cost issues, and so also was the lack of highly sophisticated quantifications, such as the volumetric wear measurement of the tapers [21]. For future longer tests with larger sample sizes, modifications to our HJS were designed so that 12 instead of 3 taper connections can be tested simultaneously (Figures 2E, 2F, and 2G). No test method, however rigorous, can fully predict the in vivo performance in complex biomechanical conditions. Hence, laboratory results need to be compared with retrieval observations when they become available.

We emphasize that our study was limited to 2 designs with Ti alloy stems and to long-neck heads available for them. The results we obtained may not be readily generalized to other designs with their long-neck heads.

### Conclusion

We showed that the wear performance of long and extra-long head taper connections showed increased wear compared with that of medium heads. ZTA heads exhibited better resistance to tribological surface degradation compared with the CoCr heads under equivalent conditions. Long necks increased taper wear and overall metal release.

*In perspective,* our findings suggest that increased neck lengths may increase micromotion and wear, and with CoCr heads they may give cause for concern. These findings should be evaluated in larger studies to determine their long-term wear performance and evaluate their clinical significance.

## Notes

MRdS: conceptualization, data curation, formal analysis, methodology, investigation, validation, visualization, writing—original draft, writing—review and editing. PN: conceptualization, writing—review and editing. VS: conceptualization, data curation, formal analysis, funding acquisition, methodology, investigation, project administration, supervision, validation, visualization, writing—original draft, writing—review and editing.

Handling co-editor: Marianne Westberg

*Acta* thanks Paul Johan Høl and other anonymous reviewers for help with peer review of this manuscript.
